# Evaluation of Q-linea ASTar rapid phenotypic antimicrobial susceptibility testing and potential impact in patients with gram-negative bloodstream infections

**DOI:** 10.1128/spectrum.03917-25

**Published:** 2026-06-15

**Authors:** Elizabeth M. Garrett, Mark D. Lesher, Evgenii Kovtunov, Cory M. Hale, Meng Pu, April M. Bobenchik

**Affiliations:** 1 Department of Pathology and Laboratory Medicine, Division of Clinical Pathology, Penn State College of Medicine 12310 , Hershey, Pennsylvania, USA; 2 Department of Pharmacy Services, Penn State Health Milton S. Hershey Medical Center, Hershey, Pennsylvania, USA; NHLS Tygerberg/Stellenbosch University, Cape Town, Western Cape, South Africa

**Keywords:** antimicrobial stewardship, bacteremia, rapid antibiotic susceptibility testing, bloodstream infections, antibiotic susceptibility testing, sepsis

## Abstract

Rapid diagnostics can drive antimicrobial stewardship strategies and allow clinicians to develop more precise and individualized treatment pathways. Q-linea ASTar is an FDA-cleared *in vitro* diagnostic for rapid phenotypic antimicrobial susceptibility testing (AST) system in patients with gram-negative blood stream infections (BSIs). In this study, we report the performance of Q-linea ASTar compared with the standard of care (SOC) method, MicroScan. A total of 83 clinical samples were enrolled in the study. The overall categorical agreement (CA) after discrepancy analysis was 94.6%, with 0.5% very major errors (1/218; VME) and 0.7% major errors (9/1,282; ME). In addition to assay performance, we assessed the hypothetical impact of rapid AST on antimicrobial stewardship prescribing practices for 57 patients with gram-negative BSI. Twenty-nine patients (50.9%) had a potential impact from ASTar on antimicrobial management, including 27 instances of de-escalation. For patients with potential impact, targeted therapy could be achieved in 22.7 h (IQR 21.0-26.3; *P* < 0.001) from blood culture collection, with a median time savings of 41.8 h compared with SOC. In summary, Q-linea ASTar performed favorably to SOC methods for AST and demonstrates the potential for shorter time to targeted antibiotic therapy in patients with gram-negative BSI.

**IMPORTANCE:**

Rapid phenotypic antimicrobial susceptibility testing (AST) has the potential to have significant impacts on antimicrobial stewardship and the treatment of blood stream infections (BSIs). There is a gap in information regarding the optimal utilization and clinical impact of rapid AST. This study evaluates new technology in the field of rapid AST, including its performance compared with standard of care practices and its potential impacts on the use of antibiotics in patients with gram-negative BSIs.

## INTRODUCTION

Bloodstream infections (BSIs) are a leading cause of mortality in North America and Europe, with the incidence of BSI continuing to rise (1–3). Gram-negative bacteria are estimated to cause over 40% of BSI (4, 5). Faster time to effective therapy improves outcomes such as mortality and length of stay in patients with bacteremia and sepsis (6, 7). Rising antibiotic resistance among gram-negative organisms may contribute to delays in effective therapy for patients with bacteremia (4, 8). To achieve early effective therapy, clinicians may use broad-spectrum antibiotics, such as carbapenems (9). However, this may increase the risk of antibiotic-associated adverse effects, such as drug toxicities or *Clostridium difficile* infection, in addition to contributing to the development of antibiotic resistance (10–13). Achieving narrow, targeted therapy may help to reduce the risk of antibiotic-associated adverse effects and impact outcomes, such as length of stay and mortality (14–17). Optimization of antibiotic therapy is a key initiative of antibiotic stewardship to fight against the rising incidence of antibiotic resistance and to improve patient outcomes.

The balance between quickly achieving effective therapy versus narrow therapy is hindered by delays in diagnostic testing. The time from blood culture collection to antibiotic susceptibility testing (AST) results can take more than 3 days due to multiple steps requiring sufficient bacterial growth. Conventional AST methods, such as disk diffusion or broth microdilution, require 16–24 h of growth before results can be interpreted. Commercially available molecular methods can shorten the time to bacterial identification and detection of antibiotic resistance mechanisms by directly testing from positive blood cultures (18, 19). Such methods, especially in combination with antibiotic stewardship efforts, can decrease the time to effective therapy and de-escalation of antimicrobials and are recommended by the Infectious Diseases Society of America (IDSA) and the Society for Healthcare Epidemiology of America (20–22). However, molecular detection of antibiotic resistance is somewhat limited in gram-negative organisms due to the wide diversity of genes causing resistance, the combinatorial effects of multiple factors, and differences in gene expression (23).

To address these limitations, there has been a need for the development of phenotypic AST methods performed directly from blood cultures. Standardized methods for disk diffusion from positive blood cultures have been published by both the Clinical and Laboratory Standards Institute (CLSI) and the European Committee on Antimicrobial Susceptibility Testing (EUCAST); however, these methods cover limited antibiotics and species (24, 25). Commercial rapid AST systems have been approved for *in vitro* diagnostic (IVD) use in Europe and the United States. These commercial devices use a variety of methods for detecting early impact of antibiotics on bacterial growth, including microscopic morphokinetic analysis, detection of volatile compounds, or use of microfluidic sensors to measure cell mass (21, 26–28). Studies consistently demonstrate a shorter time to results for rapid AST methods compared with conventional methods (21, 26, 27). Additionally, some studies demonstrate promising impacts on time to effective therapy (29–31).

Q-linea ASTar is an automated system for phenotypic AST directly from blood cultures that generates results in 6–7 h for gram-negative organisms (32). The ASTar system generates minimum inhibitory concentration (MIC) results using time-lapse microscopy to assess bacterial growth at different antibiotic concentrations in a panel format. Here we compare the performance of ASTar to MicroScan at an academic hospital laboratory. Additionally, we performed a hypothetical analysis to investigate whether ASTar results could have facilitated a therapeutic change earlier than the current standard of care (SOC) methods, evaluating which patients were likely to be impacted and the estimated time it would take to achieve targeted therapy.

## MATERIALS AND METHODS

### Study design

This study was conducted at Penn State Milton S. Hershey Medical Center, a 611-bed academic medical center located in central Pennsylvania. Consecutive positive blood cultures with gram-negative bacilli and positive for Enterobacterales*, Pseudomonas aeruginosa, Acinetobacter baumannii*, or *Haemophilus influenzae* were enrolled in the study.

This study consisted of two parts. Part 1 was the evaluation of the Q-linea ASTar BC G- panel compared with the MicroScan NM56 panel. Categorical agreement, essential agreement, and error rates were calculated for ASTar, and reference broth microdilution was used for discrepancy analysis. Due to the low number of resistant isolates, contrived samples were included in the comparison study. Part 2 of the study consisted of a retrospective chart review of the enrolled patients in part 1 to determine if the ASTar results could have led to a change in antibiotic therapy.

### Laboratory standard of care procedures

Blood cultures were collected using BACTEC Plus Aerobic/F and Lytic 10/Anaerobic/F media and incubated on the BACTEC FX automated blood culture system (BD). Following positivity, the culture was Gram-stained, and a BioFire FilmArray BCID2 (bioMérieux) panel was performed. BCID2 was performed on day and evening shifts, approximately 7 am–11 pm. BCID2 results with resistance markers such as CTX-M detected were communicated directly to pharmacy residents, who can order the first dose of an active antimicrobial agent when appropriate and follow up with clinical teams for additional management. Blood cultures were subcultured to routine microbiologic media and incubated overnight at 37°C. Matrix-assisted laser desorption/ionization-time of flight (MALDI-TOF) performed on MALDI Biotyper Sirius or Microflex (Bruker) was used for confirmation of bacterial identification. AST was performed using MicroScan NM56 panels on the MicroScan WalkAway 96 (Beckman Coulter) per manufacturer instructions using an 18–24-h subculture (33). AST results were reported using LabPro (version 4) with interpretations according to CLSI M100 (Table S1) (34, 35). AST was only performed and reported between the hours of approximately 7 am–3:30 p.m.

### ASTar AST

ASTar BC G- panels (Q-linea) were performed within 16 h of positivity of the blood cultures according to manufacturer instructions (36). Briefly, approximately 1 mL of positive blood culture was added to a consumable cartridge and loaded onto the fully automated instrument with the panel containing antibiotic dilutions in disk form. The system generates a report with minimum inhibitory concentration (MIC) results and susceptible/intermediate/resistant (S/I/R) interpretations once provided with the bacterial identification. On-panel organisms are listed in Table S2. ASTar results were generated using the manufacturer’s Investigation Use Only (IUO) software (version 1.6.7).

The isolates used for contrived specimens were patient isolates collected at this medical center and stored at −80°C. Contrived specimens were created by diluting 0.5 McFarland equivalent suspensions of overnight bacterial cultures in sterile saline to 1:1 × 10^6^ and adding 0.5 mL to BACTEC Plus Aerobic/F bottles containing human blood, which were then incubated to positivity.

Discrepant isolates were sent to a reference laboratory for reference method broth microdilution testing in triplicate with the modal MIC value used as the final reference MIC (37).

### AST performance analysis

The antibiotics, dilution ranges, and interpretations used are listed in Table S1. Categorical agreement (CA) was calculated as the percent agreement of ASTar interpretive categories compared to MicroScan. Categorical errors were defined as very major errors (VME; false susceptibility), major errors (ME; false resistance), or minor errors (mE; S versus susceptible dose dependent (SDD) or I; I or SDD to R disagreement between methods) (38). Essential agreement was calculated per ISO 20766-2:2021 by combining MICs from the method with the longer dilution range (ASTar), such that it matches those of the method with the shorter range (MicroScan) (39). Because of the large difference in dilution ranges between the two platforms, this may overestimate the EA. Bias was also calculated per ISO 20766-2:2021. A positive bias indicates that the test method MICs trend higher than the reference, and a negative bias indicates the inverse. ISO 20766-2:2021 recommends an acceptable bias within ±30%. Amikacin, tigecycline, and meropenem/vaborbactam were excluded from the bias calculation due to a lack of MICs above the lowest on-scale dilution.

ASTar results were manually reanalyzed using the FDA-cleared interpretations and limitations for reporting published in the instructions for use (Table S1) (36). The IFU indicates that “an alternative method of testing prior to reporting results” should be performed for several organism/antibiotic combinations; because these results would not be available in real-time for a rapid AST test, they were excluded from analysis.

### Clinical impact analysis

A retrospective chart review was performed to collect demographic data, laboratory results, and the time and duration of antibiotic administration. Information on the clinical course, including BSI source, was determined by reviewing the care provider’s notes, including infectious disease consult notes. Cases were reviewed by an antibiotic stewardship pharmacist to determine the first targeted therapy received by the patient and assess if there was a potential impact for intervention based on ASTar. Targeted therapy was defined as the first antimicrobial the patient received that was directed specifically at the infectious organism in accordance with institutional guidelines and with consideration for the patient’s condition, comorbidities, and infectious source. If there was a potential impact, the pharmacist determined what therapeutic change could have been made. Changes were categorized as escalation (start or addition of a broader spectrum antibiotic), de-escalation (change to a narrower spectrum antibiotic or change from intravenous (IV) to oral (PO)), or dosage adjustment.

The turnaround time (TAT) for SOC AST was calculated as the time from blood culture collection to the time SOC AST was reported in the medical record. The TAT for ASTar was calculated with the assumption that if used in routine laboratory practice, the ASTar panel would be set up at approximately the same time as the BioFire BCID2 panel, which occurs only during the day and evening shift. Thus, the TAT for ASTar was calculated as the time from the blood culture result to the time the BCID2 was started plus the actual run time for ASTar.

Time to targeted therapy (TTTT) was calculated for patients deemed to have a potential clinical impact from ASTar. SOC TTTT was calculated as the time from collection of the blood culture to the time of the first dose of the antimicrobial, determined to be the first targeted therapy received in real time by the patient. The hypothetical TTTT using ASTar was calculated as well using previously described methodology (40). For patients who achieved targeted therapy after release of SOC AST, TTTT was calculated as the TAT for ASTar plus the time between release of SOC AST to real-time TTTT. This accounts for lag time between the time AST results are available and the time the clinical team acts on them. For patients whose therapy before the release of SOC AST was appropriate as targeted therapy, TTTT for ASTar was calculated as the TAT for ASTar plus 2 h. An additional analysis was done to identify the number of monomicrobial gram-negative BSI cases across all blood cultures collected over a 6-month period.

## RESULTS

### Comparison of ASTar to MicroScan

Seventy-three patient blood cultures were prospectively enrolled in the study. Six samples were excluded from further analysis; three were found to be polymicrobial after growth of the subculture, and three failed to generate a result on ASTar. Of the 67 patient blood cultures included in the analysis, 63 were positive for Enterobacterales and 4 for *Pseudomonas aeruginosa* (Table S2). Due to a lack of resistant isolates in the study, an additional 16 contrived specimens were included using frozen isolate stocks from the laboratory. In total, there were 12 CTX-M extended spectrum β-lactamases (ESBL) and 7 carbapenem-resistant isolates.

The overall CA was 94.1%, and EA was 97.2%, with 3 VME (1.4%), 14 ME (1.09%), and 74 mE (4.8%) (Table 1). Isolates with VME and ME were tested by reference broth microdilution for discrepancy resolution (Table S3), with 47.8% (11/23) of discrepant results resolved in favor of ASTar. The CA after discrepancy analysis was 94.6%, with 1 VME (0.5%), 9 ME (0.7%), and 73 mE (4.7%). The EA after discrepancy analysis was 97.7% with 0.2% bias. The CA for Enterobacterales was 95.2%, with 1 VME (0.5%), 9 ME (0.7%), and 59 mE (4.1%) (Tables 1 and 2). Two antibiotics did not achieve ≥90% CA: ampicillin/sulbactam (75%) and cefazolin (56%). The EA for Enterobacterales was 97.7% with −0.9% bias. The CA for *P. aeruginosa* was 88.2%, with no VME or ME and 14 mE (11.8%). The EA for *P. aeruginosa* was 97.5% with 18.8% bias.

**TABLE 1:** Performance of ASTar compared with Microscan with (right) and without (left) discrepancy analysis by broth microdilution^*a*^

	Compared with Microscan	With discrepancy analysis
CA	1,455/1,546 (94.1%)	1,463/1,546 (94.6%)
EA	1,503/1,546 (97.2%)	1,510/1,546 (97.7%)
VME	3 (1.40%)	1 (0.46%)
ME	14 (1.09%)	9 (0.70%)
mE	74 (4.78%)	73 (4.72%)
S	1286	1282
I or SDD	45	46
R	215	218

^*a*^ Discrepancy analysis was not performed for all discrepant results but was performed for nine isolates, including all VME. Categorical agreement (CA); essential agreement (EA); very major error (VME); major error (ME); minor error (mE); susceptible (S); intermediate (I); resistant (R).

**TABLE 2:** Performance of ASTar relative to MicroScan with discrepancy analysis by broth microdilution^*a,c*^

	Enterobacterales									*Pseudomonas aeruginosa*									Total CA	Total EA
Antimicrobial agent	N	S	I	R	CA	EA	VME	ME	mE	N	S	I	R	CA	EA	VME	ME	mE		
Amikacin	71	71	0	0	100%	100%	0	0	0	9	9	0	0	100%	100%	0	0	0	100%	100%
Ampicillin	39	22	0	17	100%	100%	0	0	0										100%	100%
Ampicillin/ Sulbactam	59	40	10	9	75%	88%	0	2 (5%)	13 (22%)										75%	88%
Aztreonam	72	56	2	14	97%	97%	0	0	2 (3%)	11	5	1	5	73%	91%	0	0	3 (27%)	94%	96%
Cefazolin	59	37	5	17	56%	90%	0	5 (14%)	21 (36%)										56%	90%
Cefepime^*b*^	72	57	4	11	96%	97%	0	0	3 (4%)	11	6	2	3	82%	100%	0	0	2 (18%)	94%	98%
Cefotaxime	72	54	0	18	100%	97%	0	0	0										100%	97%
Ceftazidime	72	57	0	15	97%	99%	0	0	2 (3%)	11	6	0	5	100%	100%	0	0	0	98%	99%
Ceftazidime-avibactam	65	64	0	1	100%	100%	0	0	0	11	8	0	3	100%	100%	0	0	0	100%	100%
Ceftolozane-tazobactam	62	59	0	3	97%	98%	0	0	2 (3%)	11	11	0	0	91%	100%	0	0	1 (9%)	96%	99%
Ceftriaxone	72	55	1	16	100%	99%	0	0	0										100%	99%
Ciprofloxacin	72	52	3	17	96%	100%	0	0	3 (4%)	11	7	2	2	73%	91%	0	0	3 (27%)	93%	99%
Ertapenem	72	69	0	3	100%	100%	0	0	0										100%	100%
Gentamicin	72	65	2	5	99%	99%	0	0	1 (1%)										99%	99%
Levofloxacin	72	56	2	14	93%	99%	0	0	5 (7%)	11	5	3	3	73%	91%	0	0	3 (27%)	90%	98%
Meropenem	72	69	1	2	99%	97%	1 (50%)	0	0	11	6	0	5	100%	100%	0	0	0	99%	98%
Meropenem-vaborbactam	69	69	0	0	100%	100%	0	0	0										100%	100%
Piperacillin-tazobactam	72	65	3	4	94%	97%	0	0	4 (6%)	11	6	1	4	82%	100%	0	0	2 (18%)	93%	98%
Tigecycline	67	67	0	0	100%	100%	0	0	0										100%	100%
Tobramycin	72	63	4	5	94%	97%	0	1 (2%)	3 (4%)	11	11	0	0	100%	100%	0	0	0	95%	98%
Trimethoprim-sulfamethoxazole	72	55	0	17	99%	96%	0	1 (2%)	0										99%	96%
Total	1427	1202	37	188	95%	98%	1 (0.5%)	9 (0.7%)	59 (4.1%)	119	80	9	30	88%	97%	0	0	14 (12%)	95%	98%

^*a*^ Categorical agreement (CA); essential agreement (EA); very major error (VME); major error (ME); minor error (mE); susceptible (S); susceptible dose dependent (SDD); intermediate (I); resistant (R).

^*b*^ Interpretation for cefepime is S/SDD/R.

^*c*^ Gray shading indicates that no data are available for these antimicrobial agents.

At the time the study was being conducted, ASTar BC G- received FDA clearance. Using the interpretations and reporting limitations that were published in the IFU, we re-analyzed the ASTar results. After discrepancy resolution, the CA was 94.3%, and EA was 97.9%, with 1 VME (0.8%), 4 ME (0.5%), and 47 mE (5.0%) (Table S4).

### Turnaround time (TAT)

**Fig 1:**
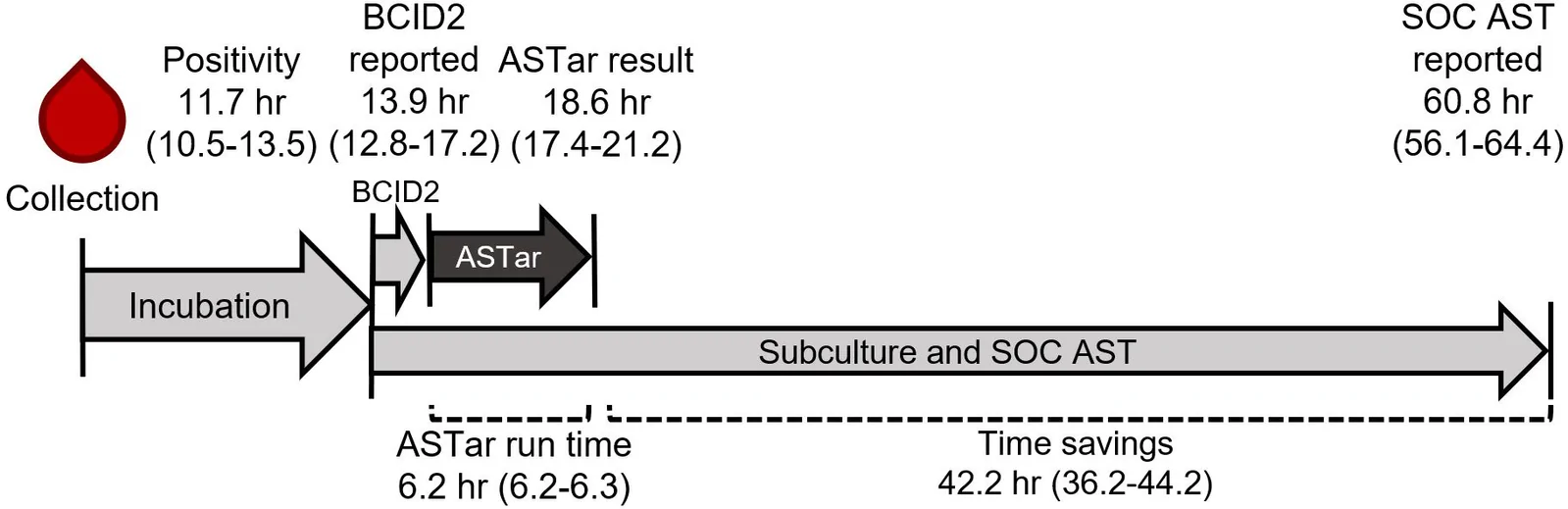
Timeline of SOC and ASTar AST results from positive blood cultures. Median time from collection and IQR are displayed.

The median time from blood culture collection to the time of the SOC AST result (turnaround time, TAT) was 60.8 h (IQR 56.1–64.4) (Fig. 1). This time includes the blood culture median growth time of 11.7 h (IQR 10.5–13.5). The median run time for ASTar was 6.2 h (IQR 6.2–6.3). We estimated the TAT for ASTar using the run time added to the time at which the BCID2 panel was performed. The median TAT for ASTar was estimated to be 18.6 h (IQR 17.4–21.2). This would result in time savings of 42.2 h (IQR 36.2–44.2).

### Potential clinical impact of rapid AST

To determine the potential impact of ASTar on the management of antibiotic therapy in patients with gram-negative BSI, a retrospective chart review was performed on the 67 patients from part 1 of the study. Ten patients were excluded from further analysis because they were not admitted, discharged, deceased, or moved to comfort care measures ≤4 days after the collection of the enrolled blood culture. For the remaining patients, the mean age was 65.1 years (range 25–87), and 27/57 (47.4%) were male (Table 3). The most common source of infection was urinary (29/57, 50.9%), followed by gastrointestinal/intra-abdominal (16/57, 28.1%). Most blood cultures were collected in the emergency department (ED) (46/57, 80.7%).

**TABLE 3:** Demographic features of subjects enrolled in this study^*a*^

	All subjects	Clinical impact	No clinical impact	*P*
Subjects	57	29	28	–^*b*^
Male	27 (47.4%)	14 (48.3%)	16 (57.1%)	0.60
Age (mean)	65.1 years	66.1 years	64.0 years	0.60
Collected in ED	46 (80.7%)	25 (86.2%)	21 (75.0%)	0.33
Admitted to ICU	11 (19.3%)	7 (24.1%)	4 (14.3%	0.50
Sepsis or septic shock	33 (57.9%)	20 (69.0%)	13 (46.4%)	0.11
Immunocompromised	13 (22.8%)	3 (10.3%)	10 (35.7%)	0.03
ID consult	24 (42.1%)	12 (41.4%)	12 (42.9%)	1.00
Potential for earlier escalation of therapy	2 (3.51%)	2 (6.9%)	–	–
Potential for earlier de-escalation of therapy	27 (47.4%)	27 (93.1%)	–	–
Presumptive source of BSI				
GI/intraabdominal	16 (28.1%)	5 (17.2%)	11 (39.3%)	0.08
Neutropenic fever hematogenous seeding	3 (5.3%)	0 (0%)	3 (10.7%)	0.11
Orthopedic/bone	3 (5.3%)	3 (10.3%)	0 (0%)	0.24
Pulmonary	3 (5.3%)	1 (3.4%)	2 (7.1%)	0.61
Skin/soft tissue	1 (1.8%)	1 (3.4%)	0 (0%)	1.00
Urinary/urogenital	29 (50.9%)	18 (62.1%)	11 (39.3%)	0.11
Unknown/other	2 (3.5%)	1 (3.4%)	1 (3.6%)	1.00

^*a*^ The "Clinical Impact" group refers to subjects with a possible impact of rapid AST on antibiotic therapy. Statistical analysis using T-test (age) or Fisher’s exact test to determine significance between the clinical impact and no impact groups. Emergency department (ED); intensive care unit (ICU); infectious diseases (ID); bloodstream infection (BSI).

^*b*^ “-” indicates not applicable.

Subjects were categorized as having or not having a potential for clinical impact due to ASTar. Potential impacts included escalation, de-escalation, or adjusted dosing of antimicrobial therapy that could have occurred with rapid AST results available after blood culture positivity and before SOC AST results. Clinical characteristics of the study population are listed in Table 3. Twenty-nine patients (50.9%) were identified as having a potential for changed management of antimicrobials given the availability of a rapid AST result. There was no significant difference in age or sex between subjects in the potential impact and no impact groups. The most common presumptive source for BSI among patients with a potential clinical impact from ASTar was urinary/urogenital (18/29 (62.1%)). Of the 29 patients for which ASTar may have impacted therapy, 27 (93.1%) may have been able to de-escalate therapy with rapid AST before SOC AST, and 2 (6.9%) may have prompted escalated therapy. The hypothetical time-to-targeted therapy (TTTT) calculated for patients with a potential impact from ASTar was 22.7 h (IQR 21.0–26.3). The real-time TTTT for the same patients was 66.7 h (IQR 59.0–69.8). This would result in median time savings of 41.8 h (IQR 36.3–44.1).

Overall, 28/57 (49.1%) subjects included in this analysis were evaluated as unlikely to have an impact from ASTar rapid AST on therapy. The most common source among the patients with no potential impact was GI/intrabdominal (11/28, 39.3%). The most common reason was that these patients were already on appropriate therapy at the time ASTar results would have been available (12/28, 42.9%). Other reasons that subjects may not have had a benefit from rapid AST were that they had a complicated source or multiple infectious processes preventing de-escalation (7/28, 25.0%) or that the patient was immunocompromised, especially neutropenic, and broad therapy was indicated (7/28, 25.0%). A total of 10/13 (76.9%) immunocompromised patients in this study were unlikely to have a potential impact on therapy from ASTar (*P* = 0.03; Table 3)

To further assess the potential impact of rapid AST at our medical center, we estimated the number of patients would have blood cultures suitable for ASTar by reviewing all blood culture orders from a 6-month period. Within that period, there were a total of 16,382 blood culture sets collected with 8.3% positivity (Fig. S1). Moreover, 74.8% (1013/1354) of the positive cultures were polymicrobial or grew off-panel organisms, such as gram-positive bacteria, yeast, or gram-negative bacteria not indicated for use with the panel. Of the remaining cultures, 341 (2.1%) cultures from 188 unique patients were monomicrobial with on-panel organisms.

## DISCUSSION

In this study, we evaluated the performance of the Q-linea ASTar system for rapid AST compared with MicroScan WalkAway. The Q-linea ASTar performed very well with a CA of ≥90% and <3% VME and ME. Discrepancy analysis resolved approximately equally in favor of ASTar or MicroScan. The results shown here are similar to those found in other studies comparing ASTar to other commercial systems (32, 41). The two antibiotics that did not achieve CA of ≥90% were ampicillin/sulbactam and cefazolin. The CA of 56% for cefazolin was lower than previously described (32). When the FDA-cleared version of the software was applied, the performance of the panel was similar to that of the IUO interpretations. However, the FDA cleared software applied significant limitations on what results could be reported without requiring a confirmatory method, and select drugs were not cleared for reporting (e.g. ceftriaxone), necessitating validation as a laboratory-developed test.

Benefits of the ASTar system include the fast turnaround time, with most results received in less than 6.5 h. The system is easy to use, with minimal hands-on time. The system can load up to six gram-negative isolates at a time and run up to 12 susceptibility discs simultaneously. The BC G- panel has a broad range of antibiotics. Additionally, it has wider dilution ranges than many commercial panels. This can be beneficial for laboratories when implementing breakpoint changes. Because this system does not include organism identification, laboratories will have to adopt a method of direct from blood culture identification to use this system. A challenge for rapid AST systems for use with blood cultures is that cultures may not be identified as polymicrobial; for example, within this study, three cultures contained multiple enteric gram-negative rods not distinguishable by Gram stain and not identified as polymicrobial by the BCID2 molecular panel. ASTar results were generated, and erroneous results could have been released prior to recognition that the culture was polymicrobial from subculture plates. Similar challenges have been seen with rapid AST systems in other studies and may negatively impact patients (21, 42).

We also performed a hypothetical analysis to determine how ASTar could have impacted antibiotic therapy for patients with gram-negative BSI. We found that approximately half of the patients evaluated had the potential for adjustment of antibiotic therapy given the availability of rapid AST, predominantly de-escalation. This is likely impacted by the relatively low rate of resistance at our medical center, including low rates of carbapenem-resistant organisms. Additionally, our workflow already utilizes a molecular panel to identify resistance genes from positive blood cultures, the results of which would be available several hours prior to rapid phenotypic AST. Therefore, the potential for greatest impact in this setting is in earlier de-escalation of antibiotic therapy. This finding is concordant with several interventional studies demonstrating an impact for rapid AST on faster de-escalation (30, 42–44). However, studies evaluating patient outcomes with rapid AST have shown mixed impact on length of stay, and few have demonstrated impacts on other metrics such as mortality (45). Many such studies encompass diverse patient populations; identifying patients with the greatest potential impact from rapid AST could show more benefits from this technology.

The most common source of BSI in the patients in this study was urinary. Of 29 patients with a urinary source, 18 (62.1%) were judged to have a potential impact on therapy from ASTar. In contrast, only 5/16 (31.3%) of patients with an GI/intraabdominal source were likely to have impacted therapy. This may reflect that less complicated, monomicrobial sources may be more likely to be impacted by rapid AST, especially to de-escalate therapy. We found that immunocompromised or neutropenic patients were significantly less likely to have a potential impact from rapid AST. However, a study investigating the use of rapid AST in patients with hematologic malignancies still saw an impact on time to optimal therapy and reduced use of broad-spectrum antibiotics (46). Differences between these studies could be due to differences in institutional antibiotic management practices or antibiotic resistance rates. Further studies to define the utility of rapid AST on specific patient populations are needed.

ASTar system had the potential to generate results over 40 h faster than SOC methods requiring subculture. While this TAT was simulated, it was based on our real laboratory practice of performing rapid molecular panels from blood cultures during day and evening shifts. However, due to the longer run time for ASTar relative to our SOC molecular panel, this would result in some ASTar results generating in the overnight hours when AST results are not currently released. For greater benefit from rapid diagnostics, laboratories may consider expanding overnight services (47). In addition, rapid diagnostics, including rapid AST, can have a greater impact with close collaboration with antimicrobial stewardship teams in order to act quickly on rapid results (46, 48–50). Implementation of rapid AST may require significant changes to both laboratory and clinical workflows to see benefits.

Limitations of this analysis include the small study populations, comparison to a commercial system, and the retrospective nature. Although ASTar and MicroScan demonstrated high agreement, either system may demonstrate inaccuracy compared to reference method broth microdilution. Additionally, the dilution ranges between both systems were quite different (Table S1), which limited our ability to accurately assess EA and bias due to the high amount of off-scale results. The potential for impact was assessed using clinical data available at the time rapid AST would have been available and by reading contemporary notes in the medical records. However, this model of analysis may overestimate the number of patients that may have been impacted; in real time, the source for the BSI, concurrent infectious processes, and patient stability may be unclear, and this may deter clinicians from acting on rapid AST to de-escalate. Another reason clinicians may not act on rapid AST may be due to waiting for other laboratory or imaging results before de-escalation. Antimicrobial stewardship teams may play a key role in encouraging appropriate action on rapid diagnostic results and integrating new technologies into clinical practice.

The ASTar system is a new-to-market system for rapid AST that performs comparably to conventional AST systems, such as MicroScan. Advances in rapid phenotypic AST from blood cultures have the potential to positively impact patient care and reduce unnecessary antibiotic use. Utilization of rapid diagnostics represents a key opportunity for clinical microbiology laboratories and antimicrobial stewardship programs to collaborate.

## SUPPLEMENTAL MATERIAL

Supplemental material spectrum.03917-25-s0001.docx Tables S1 to S4; Fig. S1.

ASM does not own the copyrights to Supplemental Material that may be linked to, or accessed through, an article. The authors have granted ASM a non-exclusive, world-wide license to publish the Supplemental Material files. Please contact the corresponding author directly for reuse.

## References

[1] Goto M, Al-Hasan MN. 2013. Overall burden of bloodstream infection and nosocomial bloodstream infection in North America and Europe. Clin Microbiol Infect 19:501–509. doi:10.1111/1469-0691.12195 23473333

[2] Holmbom M, Giske CG, Fredrikson M, Östholm Balkhed Å, Claesson C, Nilsson LE, Hoffmann M, Hanberger H. 2016. 14-year survey in a Swedish county reveals a pronounced increase in bloodstream infections (BSI). Comorbidity - an independent risk factor for both BSI and mortality. PLoS One 11:e0166527. doi:10.1371/journal.pone.0166527 27835663 PMC5106013

[3] Kontula KSK, Skogberg K, Ollgren J, Järvinen A, Lyytikäinen O. 2021. Population-based study of bloodstream infection incidence and mortality rates, Finland, 2004–2018. Emerg Infect Dis 27:2560–2569. doi:10.3201/eid2710.204826 34546161 PMC8462341

[4] Diekema DJ, Hsueh P-R, Mendes RE, Pfaller MA, Rolston KV, Sader HS, Jones RN. 2019. The microbiology of bloodstream infection: 20-year trends from the SENTRY antimicrobial surveillance program. Antimicrob Agents Chemother 63:e00355-19. doi:10.1128/AAC.00355-19 31010862 PMC6591610

[5] Bauer KA, Puzniak LA, Yu KC, Finelli L, Moise P, Ai C, Watts JA, Gupta V. 2022. Epidemiology and outcomes of culture-positive bloodstream pathogens prior to and during the SARS-CoV-2 pandemic: a multicenter evaluation. BMC Infect Dis 22:841. doi:10.1186/s12879-022-07810-8 36368931 PMC9651895

[6] Kang C-I, Kim S-H, Park WB, Lee K-D, Kim H-B, Kim E-C, Oh M-D, Choe K-W. 2005. Bloodstream infections caused by antibiotic-resistant gram-negative bacilli: risk factors for mortality and impact of inappropriate initial antimicrobial therapy on outcome. Antimicrob Agents Chemother 49:760–766. doi:10.1128/AAC.49.2.760-766.2005 15673761 PMC547233

[7] Kumar A, Roberts D, Wood KE, Light B, Parrillo JE, Sharma S, Suppes R, Feinstein D, Zanotti S, Taiberg L, et al.. 2006. Duration of hypotension before initiation of effective antimicrobial therapy is the critical determinant of survival in human septic shock. Crit Care Med 34:1589–1596. doi:10.1097/01.CCM.0000217961.75225.E9 16625125

[8] Bassetti M, Kanj SS, Kiratisin P, Rodrigues C, Van Duin D, Villegas MV, Yu Y. 2022. Early appropriate diagnostics and treatment of MDR Gram-negative infections. JAC Antimicrob Resist 4:dlac089. doi:10.1093/jacamr/dlac089 36111208 PMC9469888

[9] Rhee C, Kadri SS, Dekker JP, Danner RL, Chen H-C, Fram D, Zhang F, Wang R, Klompas M. 2020. Prevalence of antibiotic-resistant pathogens in culture-proven sepsis and outcomes associated with inadequate and broad-spectrum empiric antibiotic use. JAMA Netw Open 3:e202899. doi:10.1001/jamanetworkopen.2020.2899 32297949 PMC7163409

[10] Miller AC, Arakkal AT, Sewell DK, Segre AM, Tholany J, Polgreen PM, CDC MInD-Healthcare Group. 2023. Comparison of different antibiotics and the risk for community-associated *Clostridioides difficile* infection: a case-control study. Open Forum Infect Dis 10:ofad413. doi:10.1093/ofid/ofad413 37622034 PMC10444966

[11] Gilboa M, Regev-Yochay G, Meltzer E, Cohen I, Peretz Y, Zilberman-Daniels T, Segev A, Amit S, Yahav D, Barda N. 2025. Antibiotic use and the risk of hospital-onset *Clostridioides difficile* infection. JAMA Netw Open 8:e2525252. doi:10.1001/jamanetworkopen.2025.25252 40779269 PMC12334957

[12] Tamma PD, Avdic E, Li DX, Dzintars K, Cosgrove SE. 2017. Association of adverse events with antibiotic use in hospitalized patients. JAMA Intern Med 177:1308–1315. doi:10.1001/jamainternmed.2017.1938 28604925 PMC5710569

[13] Teshome BF, Vouri SM, Hampton N, Kollef MH, Micek ST. 2019. Duration of exposure to antipseudomonal β-lactam antibiotics in the critically ill and development of new resistance. Pharmacotherapy 39:261–270. doi:10.1002/phar.2201 30506852 PMC6507412

[14] Lee C-C, Wang J-L, Lee C-H, Hung Y-P, Hong M-Y, Tang H-J, Ko W-C. 2017. Clinical benefits of antimicrobial de-escalation in adults with community-onset monomicrobial *Escherichia coli*, *Klebsiella* species and *Proteus mirabilis* bacteremia. Int J Antimicrob Agents 50:371–376. doi:10.1016/j.ijantimicag.2017.03.024 28694235

[15] Alanazi A, Almuhaya R, Almohaimeed M, Alahmari N, Abdulrahim N, Basyouni M, Althikrallah F, Al Badwyi J, Khallaf A, Albalawi K, et al.. 2023. Impact of antibiotic de-escalation on antibiotic consumption, length of hospitalization, mortality, and cost: a systematic review and meta-analysis. Pharmacoepidemiology 2:289–306. doi:10.3390/pharma2040025

[16] Kam KQ, Chen T, Kadri SS, Lawandi A, Yek C, Walker M, Warner S, Fram D, Chen H-C, Shappell CN, et al.. 2025. Epidemiology and outcomes of antibiotic de-escalation in patients with suspected sepsis in US hospitals. Clin Infect Dis 80:108–117. doi:10.1093/cid/ciae591 39657050 PMC11797381

[17] Teshome BF, Park T, Arackal J, Hampton N, Kollef MH, Micek ST. 2024. Preventing new gram-negative resistance through beta-lactam de-escalation in hospitalized patients with sepsis: a retrospective cohort study. Clin Infect Dis 79:826–833. doi:10.1093/cid/ciae253 38842541

[18] Tansarli GS, Chapin KC. 2022. A closer look at the laboratory impact of utilizing ePlex blood culture identification panels: a workflow analysis using rapid molecular detection for positive blood cultures. Microbiol Spectr 10:e0179622. doi:10.1128/spectrum.01796-22 36069598 PMC9602361

[19] Peri AM, Ling W, Furuya-Kanamori L, Harris PNA, Paterson DL. 2022. Performance of BioFire Blood Culture Identification 2 Panel (BCID2) for the detection of bloodstream pathogens and their associated resistance markers: a systematic review and meta-analysis of diagnostic test accuracy studies. BMC Infect Dis 22:794. doi:10.1186/s12879-022-07772-x 36266641 PMC9585790

[20] Banerjee R, Teng CB, Cunningham SA, Ihde SM, Steckelberg JM, Moriarty JP, Shah ND, Mandrekar JN, Patel R. 2015. Randomized trial of rapid multiplex polymerase chain reaction–based blood culture identification and susceptibility testing. Clin Infect Dis 61:1071–1080. doi:10.1093/cid/civ447 26197846 PMC4560903

[21] Pancholi P, Carroll KC, Buchan BW, Chan RC, Dhiman N, Ford B, Granato PA, Harrington AT, Hernandez DR, Humphries RM, et al.. 2018. Multicenter evaluation of the accelerate PhenoTest BC kit for rapid identification and phenotypic antimicrobial susceptibility testing using morphokinetic cellular analysis. J Clin Microbiol 56:e01329-17. doi:10.1128/JCM.01329-17 PMC5869823 29305546

[22] Barlam TF, Cosgrove SE, Abbo LM, MacDougall C, Schuetz AN, Septimus EJ, Srinivasan A, Dellit TH, Falck-Ytter YT, Fishman NO, et al.. 2016. Implementing an antibiotic stewardship program: guidelines by the Infectious Diseases Society of America and the Society for Healthcare Epidemiology of America. Clin Infect Dis 62:e51–e77. doi:10.1093/cid/ciw118 27080992 PMC5006285

[23] Hattab S, Ma AH, Tariq Z, Vega Prado I, Drobish I, Lee R, Yee R. 2024. Rapid phenotypic and genotypic antimicrobial susceptibility testing approaches for use in the clinical laboratory. Antibiotics (Basel) 13:786. doi:10.3390/antibiotics13080786 39200086 PMC11351821

[24] European Committee on Antimicrobial Susceptibility Testing (EUCAST). 2025. EUCAST rapid antimicrobial susceptibility testing (RAST) directly from positive blood culture bottles version 6.0

[25] Clinical and Laboratory Standards Institute. 2025. Performance standards for antimicrobial susceptibility testing. *In* CLSI supplement M100, 35th ed. Clinical and Laboratory Standards Institute (CLSI).

[26] Tibbetts R, George S, Burwell R, Rajeev L, Rhodes PA, Singh P, Samuel L. 2022. Performance of the reveal rapid antibiotic susceptibility testing system on gram-negative blood cultures at a large urban hospital. J Clin Microbiol 60:e0009822. doi:10.1128/jcm.00098-22 35607972 PMC9199398

[27] Snyder JW, Chaudhry N, Hoffmann W. 2024. Performance of the LifeScale automated rapid phenotypic antimicrobial susceptibility testing on gram-negative rods directly from positive blood cultures. J Clin Microbiol 62:e0092224. doi:10.1128/jcm.00922-24 39480069 PMC11633210

[28] Messiaen A-S, Vandendriessche S, De Muynck E, Strubbe G, De Bus L, Schelstraete P, Decommer K, De Smet S, Soetens A, Naesens L, et al.. 2025. Impact of reporting rapid susceptibility results in gram negative bloodstream infections: a real world prospective study. Eur J Clin Microbiol Infect Dis 44:847–853. doi:10.1007/s10096-025-05046-3 39862301

[29] Ventres JJ, Ting MH, Parente DM, Rogers R, Norris AM, Benitez G, Shehadeh F, Bobenchik AM, Mylonakis E, Chapin KC, et al.. 2024. Combination of a rapid diagnostic assay and antimicrobial stewardship intervention for gram-negative bacteremia. Open Forum Infect Dis 11:ofae477. doi:10.1093/ofid/ofae477 39263216 PMC11389609

[30] Ehren K, Meißner A, Jazmati N, Wille J, Jung N, Vehreschild JJ, Hellmich M, Seifert H. 2020. Clinical impact of rapid species identification from positive blood cultures with same-day phenotypic antimicrobial susceptibility testing on the management and outcome of bloodstream infections. Clin Infect Dis 70:1285–1293. doi:10.1093/cid/ciz406 31094414

[31] Banerjee R, Komarow L, Virk A, Rajapakse N, Schuetz AN, Dylla B, Earley M, Lok J, Kohner P, Ihde S, et al.. 2021. Randomized trial evaluating clinical impact of RAPid IDentification and susceptibility testing for gram-negative bacteremia: RAPIDS-GN. Clin Infect Dis 73:e39–e46. doi:10.1093/cid/ciaa528 32374822 PMC8246790

[32] Göransson J, Sundqvist M, Ghaderi E, Lisby JG, Molin Y, Eriksson E, Carlsson S, Cederlöf A, Ellis L, Melin J. 2023. Performance of a system for rapid phenotypic antimicrobial susceptibility testing of gram-negative bacteria directly from positive blood culture bottles. J Clin Microbiol 61:e0152522. doi:10.1128/jcm.01525-22 36852983 PMC10035315

[33] MicroScan gram negative procedural manual, LabPro ≥V4.42 PU-08 panel C42772-AB. 2019. Beckman Coulter

[34] Clinical and Laboratory Standards Institute. 2022. Performance standards for antimicrobial susceptibility testing. *In* CLSI supplement M100, 32rd ed. Clinical and Laboratory Standards Institute (CLSI).

[35] Clinical and Laboratory Standards Institute. 2024. Performance standards for antimicrobial susceptibility testing. *In* , 34th ed. Clinical and Laboratory Standards Institute (CLSI).

[36] Q-linea. 2024. ASTar BC G− Kit Instructions for use version 6

[37] Clinical and Laboratory Standards Institute. 2024. Methods for dilution antimicrobial susceptibility tests for bacteria that grow aerobically. *In* M07, 12th ed. Clinical and Laboratory Standards Institute (CLSI).

[38] Clinical and Laboratory Standards Institute. 2018. Development of *in vitro* susceptibility testing criteria and quality control parameters. *In* CLSI supplement M23, 5th ed. Clinical and Laboratory Standards Institute.

[39] ISO 20776-2:2021(E) Clinical laboratory testing and *in vitro* diagnostic test systems — Susceptibility testing of infectious agents and evaluation of performance of antimicrobial susceptibility test devices — Part 2: evaluation of performance of antimicrobial susceptibility test devices against reference broth microdilution. 2021

[40] Yuceel-Timur I, Thierry E, Chainier D, Ndao I, Labrousse M, Grélaud C, Bala Y, Barraud O. 2024. Retrospective evaluation of rapid genotypic ID and phenotypic AST systems on positive blood culture turnaround time and simulated potential impacts on bloodstream infection management. J Antimicrob Chemother 79:i26–i31. doi:10.1093/jac/dkae280 39298362 PMC11412238

[41] Esse J, Träger J, Valenza G, Bogdan C, Held J. 2023. Rapid phenotypic antimicrobial susceptibility testing of gram-negative rods directly from positive blood cultures using the novel Q-linea ASTar system. J Clin Microbiol 61:e0054923. doi:10.1128/jcm.00549-23 37819072 PMC10662367

[42] Brosh-Nissimov T, Tzur A, Grupel D, Cahan A, Ma’aravi N, Heled-Akiva M, Jawamis H, Leskes H, Barenboim E, Sorek N. 2023. Clinical impact of the accelerate PhenoTest BC system on patients with gram-negative bacteremia and high risk of antimicrobial rClinical impact of the accelerate PhenoTest BC system on patients with gram-negative bacteremia and high risk of antimicrobial resistance: a prospective before-after implementation studyesistance: a prospective before-after implementation study. Ann Clin Microbiol Antimicrob 22:62. doi:10.1186/s12941-023-00619-6 37516885 PMC10387206

[43] Truong TT, Mongkolrattanothai K, Flores II, Dien Bard J. 2022. Evaluation of the performance and clinical impact of a rapid phenotypic susceptibility testing method directly from positive blood culture at a pediatric hospital. J Clin Microbiol 60:e0012222. doi:10.1128/jcm.00122-22 35852363 PMC9383260

[44] Bhalodi AA, MacVane SH, Ford B, Ince D, Kinn PM, Percival KM, Bremmer DN, Carr DR, Walsh TL, Bhatti MM, et al.. 2022. Real-world impact of the accelerate PhenoTest BC kit on patients with bloodstream infections in the improving outcomes and antimicrobial stewardship study: a quasiexperimental multicenter study. Clin Infect Dis 75:269–277. doi:10.1093/cid/ciab921 34718456 PMC9410719

[45] MacVane SH, Dwivedi HP. 2024. Evaluating the impact of rapid antimicrobial susceptibility testing for bloodstream infections: a review of actionability, antibiotic use and patient outcome metrics. J Antimicrob Chemother 79:i13–i25. doi:10.1093/jac/dkae282 39298359 PMC11412245

[46] Kim J-H, Kim I, Kang CK, Jun K-I, Yoo SH, Chun JY, Jung J, Kim YJ, Kim DY, Jo HB, et al.. 2021. Enhanced antimicrobial stewardship based on rapid phenotypic antimicrobial susceptibility testing for bacteraemia in patients with haematological malignancies: a randomized controlled trial. Clin Microbiol Infect 27:69–75. doi:10.1016/j.cmi.2020.03.038 32272171

[47] Ingram PR, Barrett L, Raby E, Boan P, Weaire-Buchanan GA, Darragh H, Lloyd P, Kay I, Flexman J. 2021. Laboratory and clinical impacts of an overnight laboratory service. Eur J Clin Microbiol Infect Dis 40:353–359. doi:10.1007/s10096-019-03737-2 32960364

[48] Claeys KC, Heil EL, Hitchcock S, Johnson JK, Leekha S. 2020. Management of gram-negative bloodstream infections in the era of rapid diagnostic testing: impact with and without antibiotic stewardship. Open Forum Infect Dis 7:ofaa427. doi:10.1093/ofid/ofaa427 33134414 PMC7585329

[49] Timbrook TT, Morton JB, McConeghy KW, Caffrey AR, Mylonakis E, LaPlante KL. 2017. The effect of molecular rapid diagnostic testing on clinical outcomes in bloodstream infections: a systematic review and meta-analysis. Clin Infect Dis 64:15–23. doi:10.1093/cid/ciw649 27678085

[50] Babowicz F, LaPlante R, Mitchell C, O’Donnell JN, Tobin E, George M, Carreno JJ. 2021. Impact of accelerate Pheno and BacT/Alert Virtuo on clinical processes and outcomes in patients with sepsis and concurrent gram-negative bacteremia. Antimicrob Agents Chemother 65:e02364-20. doi:10.1128/AAC.02364-20 33753337 PMC8315910

