# Supplementary material for: Evaluation of Q-linea ASTar rapid phenotypic antimicrobial susceptibility testing and potential impact in patients with gram-negative bloodstream infections
Source: Microbiol Spectr. 2026 Jun 15;14(7):e03917-25. doi: 10.1128/spectrum.03917-25 (PMC13340051; doi:10.1128/spectrum.03917-25)
Supplement: Supplemental material — Tables S1 to S4; Fig. S1. [file spectrum.03917-25-s0001.docx]

| **Antimicrobial Agent** | **ASTar BC G- (IUO)** | | | | | **Microscan NM56 (IVD with lab modifications)** | | | | | | |
| --- | --- | --- | --- | --- | --- | --- | --- | --- | --- | --- | --- | --- |
|  | **Dilution (µg/mL)** |  | **S** | **I** | **R** | **Dilution (µg/mL)** | |  | **S** | **SDD** | **I** | **R** |
| Amikacin | 2-128 | En | ≤ 16 | 32 | ≥ 64 | 16-32 | | En | ≤ 16 | - | 32 | >32 |
|  |  | Ps | ≤ 16 | 32 | ≥ 64 |  |  | Ps | ≤ 16 | - | 32 | >32 |
| Ampicillin | 1-64 | En | ≤ 8 | 16 | ≥ 32 | 8-16 | | En | ≤ 8 | - | 16 | >16 |
|  |  | Ps | - | | |  |  | Ps | - | | | |
| Ampicillin/ Sulbactam | 1-64 | En | ≤ 8 | 16 | ≥ 32 | 4/2-16/8 | | En | ≤ 8/4 | - | 16/8 | >16/8 |
|  |  | Ps | - | | |  |  | Ps | - | | | |
| Aztreonam | 0.25-64 | En | ≤ 4 | 8 | ≥ 16 | 4-16 | | En | ≤ 4 | - | 8 | ≥ 16 |
|  |  | Ps | ≤ 8 | 16 | ≥ 32 |  |  | Ps | ≤ 8 | - | 16 | ≥ 32 |
| Cefazolin | 0.25-16 | En | ≤ 2 | 4 | ≥ 8 | 2-16 | | En | ≤ 2 | - | 4 | ≥ 8 |
|  |  | Ps | - | | |  |  | Ps | - | | | |
| Cefepime | 0.25-64 | En | ≤ 2 | 4, 8 | ≥ 16 | 2-16 | | En | ≤ 2 | 4, 8 | - | ≥ 16 |
|  |  | Ps | ≤ 8 | - | ≥ 16 |  |  | Ps | ≤ 8 | - | 16 | ≥ 32 |
| Cefotaxime | 0.015-128 | En | ≤ 1 | 2 | ≥ 4 | 2,8-32 | | En | ≤ 1 | - | 2 | ≥ 4 |
|  |  | Ps | - | | |  |  | Ps | - | | | |
| Cefoxitin | Excluded | | | | | | | | | | | |
| Ceftazidime | 0.25-64 | En | ≤ 4 | 8 | ≥ 16 | 1,4-16 | En | | ≤ 4 | - | 8 | ≥ 16 |
|  |  | Ps | ≤ 8 | - | ≥ 16 |  | Ps | | ≤ 8 | - | 16 | ≥ 32 |
| Ceftazidime-avibactam | 0.125-32 | En | ≤ 8 | - | ≥ 16 | 4/4-16/4 | En | | ≤ 8/4 | - | - | ≥ 16/4 |
|  |  | Ps | ≤ 8 | - | ≥ 16 |  | Ps | | ≤ 8/4 | - | - | ≥ 16/4 |
| Ceftolozane-tazobactam | 0.25-32 | En | ≤ 2 | 4 | ≥ 8 | 2/4-8/4 | En | | ≤ 2/4 | - | 4/4 | ≥ 8/4 |
|  |  | Ps | ≤ 4 | 8 | ≥ 16 |  | Ps | | ≤ 4/4 | - | 8/4 | ≥ 16/4 |
| Ceftriaxone | 0.06-128 | En | ≤ 1 | 2 | ≥ 4 | 1-2,8,32 | En | | ≤ 1 | - | 2 | ≥ 4 |
|  |  | Ps | - | | |  | Ps | | - | | | |
| Cefuroxime | Excluded | | | | | | | | | | | |
| Ciprofloxacin | 0.125-8 | En | ≤ 0.25 | 0.5 | ≥ 1 | 0.25-2 | En | | ≤ 0.25 | - | 0.5 | ≥ 1 |
|  |  | Ps | ≤ 0.5 | 1 | ≥ 2 |  | Ps | | ≤ 0.5 | - | 1 | ≥ 2 |
| Ertapenem | 0.015-4 | En | ≤ 0.5 | 1 | ≥ 2 | 0.5-1 | En | | ≤ 0.5 | - | 1 | ≥ 2 |
|  |  | Ps | - | | |  | Ps | | - | | | |
| Gentamicin | 0.25-32 | En | ≤ 4 | 8 | ≥ 16 | 2-8 | En | | ≤ 4 | - | 8 | >8 |
|  |  | Ps | Excluded | | |  | Ps | | Excluded | | | |
| Levofloxacin | 0.125-16 | En | ≤ 0.5 | 1 | ≥ 2 | 0.5-4 | En | | ≤ 0.5 | - | 1 | ≥ 2 |
|  |  | Ps | ≤ 1 | 2 | ≥ 4 |  | Ps | | ≤ 1 | - | 2 | ≥ 4 |
| Meropenem | 0.06-64 | En | ≤ 1 | 2 | ≥ 4 | 1-8 | En | | ≤ 1 | - | 2 | ≥ 4 |
|  |  | Ps | ≤ 2 | 4 | ≥ 8 |  | Ps | | ≤ 2 | - | 4 | ≥ 8 |
| Meropenem-vaborbactam | 0.25-32 | En | ≤ 4 | 8 | ≥ 16 | 2/8-16/8 | En | | ≤ 4/4 | - | 8/8 | ≥ 16/8 |
|  |  | Ps | - | | |  | Ps | | - | | | |
| Piperacillin-tazobactam* | 0.25-256 | En | ≤ 16 | 32, 64 | ≥ 128 | 8/4-64/4 | En | | ≤ 16/4 | - | 32/4, 64/4 | ≥ 128/4 |
|  |  | Ps | ≤ 16 | 32, 64 | ≥ 128 |  | Ps | | ≤ 16/4 | - | 32/4, 64/4 | ≥ 128/4 |
| Tigecycline | 0.03-16 | En | ≤ 2 | 4 | ≥ 8 | 2-4 | En | | ≤ 2 | - | 4 | >4 |
|  |  | Ps | - | | |  | Ps | | - | | | |
| Tobramycin | 1-32 | En | ≤ 4 | 8 | ≥ 16 | 2-8 | En | | ≤ 4 | - | 8 | >8 |
|  |  | Ps | ≤ 4 | 8 | ≥ 16 |  | Ps | | ≤ 4 | - | 8 | >8 |
| Trimethoprim-sulfamethoxazole | 0.06-8 | En | ≤ 2 | - | ≥ 4 | 0.5/9.5-2/38 | En | | ≤ 2/38 | - | - | ≥ 4/76 |
|  |  | Ps | - | | |  | Ps | | - | | | |

Table S1. Dilution range and interpretive breakpoints used in the evaluation.*Re-analysis using the ASTar BC GN- IVD software changed the piperacillin-tazobactam breakpoints to ≤8 S, 16 SDD, ≥ 32 R for Enterobacterales and ≤16 S, 32 I, ≥ 64 R for *P. aeruginosa*; all other antimicrobial interpretations remained the same. IUO: Investigational Use Only, IVD: Investigational Device, En: Enterobacterales, Ps: *Pseudomonas aeruginosa*.

|  | **Prospective** | **Contrived** | **Total** |
| --- | --- | --- | --- |
| *Citrobacter freundii* | 0 | 1 | 1 |
| *Enterobacter cloacae* | 4 | 2 | 6 |
| *Escherichia coli* | 33 | 1 | 34 |
| *Klebsiella aerogenes* | 2 | 0 | 2 |
| *Klebsiella oxytoca* | 3 | 1 | 4 |
| *Klebsiella pneumoniae group* | 14 | 2 | 16 |
| *Proteus mirabilis* | 3 | 2 | 5 |
| *Serratia marcescens* | 4 | 0 | 4 |
| *Pseudomonas aeruginosa* | 4 | 7 | 11 |
| **Total** | **67** | **16** | **83** |

Table S2. Isolates included in this study. The following isolates are indicated for use with the ASTar BC GN- panel, but were not included in this study; *Acinetobacter baumannii, Haemophilus influenzae, Citrobacter koserii, Morganella morganii*, and *Proteus vulgaris*

| **Antibiotic** | **Organism** | **BMD** | **MicroScan** | **ASTar** | **Error vs MicroScan** | **Error vs BMD** |
| --- | --- | --- | --- | --- | --- | --- |
| Ampicillin/sulbactam | *E. coli* | S | S | I | mE | mE |
|  | *K. pneumoniae* | S | S | R | ME | ME |
|  | *K. pneumoniae* | R | I | R | mE | no error |
| Aztreonam | *K. pneumoniae* | R | I | R | mE | no error |
| Cefazolin | *P. mirabilis* | I | S | R | ME | mE |
| Cefepime | *K. pneumoniae* | SDD | SDD | R | mE | mE |
|  | *K. pneumoniae* | SDD | SDD | R | mE | mE |
| Ceftazidime/avibactam | *P. aeruginosa* | R | S | R | ME | no error |
|  | *P. aeruginosa* | R | S | R | ME | no error |
| Ceftriaxone | *P. mirabilis* | S | R | S | VME | no error |
| Ciprofloxacin | *K. pneumoniae* | I | S | R | ME | mE |
|  | *K. pneumoniae* | R | I | R | mE | no error |
|  | *P. aeruginosa* | S | S | I | mE | mE |
| Levofloxacin | *E. coli* | I | I | R | mE | mE |
|  | *K. pneumoniae* | S | S | I | mE | mE |
|  | *K. pneumoniae* | I | S | I | mE | no error |
|  | *P. aeruginosa* | I | I | R | mE | mE |
| Meropenem | *C. freundii* | R | R | S | VME | VME |
| Piperacillin/tazobactam | *K. pneumoniae* | I | S | R | ME | mE |
|  | *P. aeruginosa* | R | R | I | mE | mE |
| Tobramycin | *E. coli* | S | S | R | ME | ME |
| Trimethoprim-sulfamethoxazole | *P. mirabilis* | S | S | R | ME | ME |
|  | *K. pneumoniae* | S | R | S | VME | no error |

Table S3. Results of discrepant analysis. Broth microdilution (BMD) was performed in triplicate for nine isolates. Very major error (VME); major error (ME); minor error (mE); susceptible (S); susceptible dose dependent (SDD); intermediate (I); resistant (R).

|  | **Enterobacterales** | | | | | | | | | ***Pseudomonas aeruginosa*** | | | | | | | | | **Total CA** | **Total EA** |
| --- | --- | --- | --- | --- | --- | --- | --- | --- | --- | --- | --- | --- | --- | --- | --- | --- | --- | --- | --- | --- |
| **Antimicrobial Agent** | N | S | I | R | CA | EA | VME | ME | mE | N | S | I | R | CA | EA | VME | ME | mE |  |  |
| Amikacin | 37 | 37 | 0 | 0 | 100% | 100% | 0 | 0 | 0 | 9 | 9 | 0 | 0 | 100% | 100% | 0 | 0 | 0 | 100% | 100% |
| Ampicillin | 39 | 22 | 0 | 17 | 100% | 100% | 0 | 0 | 0 |  | | | | | | | | | 100% | 100% |
| Ampicillin/ Sulbactam | 59 | 40 | 10 | 9 | 75% | 88% | 0 | 2 (5%) | 13 (22%) |  |  |  |  |  |  |  |  |  | 75% | 88% |
| Aztreonam | 71 | 56 | 2 | 13 | 97% | 97% | 0 | 0 | 2 | Perform an alternative method of testing prior to reporting results | | | | | | | | | 97% | 97% |
| Cefazolin | 20 | 12 | 0 | 8 | 55% | 95% | 0 | 1 (8%) | 8 (40%) |  | | | | | | | | | 55% | 95% |
| Cefepime | 62 | 48 | 4 | 10 | 95% | 97% | 0 | 0 | 3 (5%) | 11 | 6 | 2 | 3 | 82% | 100% | 0 | 0 | 2 (18%) | 92% | 97% |
| Cefotaxime | Perform an alternative method of testing prior to reporting results | | | | | | | | |  | | | | | | | | |  | |
| Ceftazidime | 35 | 28 | 0 | 7 | 94% | 97% | 0 | 0 | 2 (6%) | Perform an alternative method of testing prior to reporting results | | | | | | | | | 94% | 97% |
| Ceftazidime-avibactam | 15 | 15 | 0 | 0 | 100% | 100% | 0 | 0 | 0 | 11 | 8 | 0 | 3 | 100% | 100% | 0 | 0 | 0 | 100% | 100% |
| ceftolozane-tazobactam | Perform an alternative method of testing prior to reporting results | | | | | | | | | Perform an alternative method of testing prior to reporting results | | | | | | | | |  | |
| Ceftriaxone |  | | | | | | | | |  | | | | | | | | |  |  |
| Ciprofloxacin | 71 | 52 | 3 | 16 | 96% | 100% | 0 | 0 | 3 (4%) | 11 | 7 | 2 | 2 | 72% | 91% | 0 | 0 | 3 (27%) | 93% | 99% |
| Ertapenem |  | | | | | | | | |  | | | | | | | | |  | |
| Gentamicin | 30 | 29 | 1 | 0 | 100% | 100% | 0 | 0 | 0 |  |  |  |  |  |  |  |  |  | 100% | 100% |
| Levofloxacin | 72 | 56 | 2 | 14 | 93% | 99% | 0 | 0 | 5 (7%) | 11 | 5 | 3 | 3 | 72% | 91% | 0 | 0 | 3 (27%) | 90% | 98% |
| Meropenem | 44 | 42 | 0 | 2 | 98% | 95% | 1 (50%) | 0 | 0 | 11 | 6 | 0 | 5 | 100% | 100% | 0 | 0 | 0 | 98% | 96% |
| Meropenem-vaborbactam | 69 | 69 | 0 | 0 | 100% | 100% | 0 | 0 | 0 |  | | | | | | | | | 100% | 100% |
| Piperacillin-tazobactam | 53 | 49 | 1 | 3 | 100% | 100% | 0 | 0 | 0 |  |  |  |  |  |  |  |  |  | 100% | 100% |
| Tigecycline | 67 | 67 | 0 | 0 | 100% | 100% | 0 | 0 | 0 |  |  |  |  |  |  |  |  |  | 100% | 100% |
| Tobramycin | 66 | 57 | 4 | 5 | 94% | 98% | 0 | 1 (2%) | 3 (5%) |  |  |  |  |  |  |  |  |  | 94% | 98% |
| Trimethoprim-sulfamethoxazole | 57 | 48 | 0 | 9 | 98% | 98% | 0 | 0 | 0 |  |  |  |  |  |  |  |  |  | 98% | 98% |
| **Total** | 867 | 727 | 27 | 113 | 95% | 98% | 1 (0.9%) | 4 (0.6%) | 39 (4.5%) | 64 | 41 | 7 | 16 | 88% | 97% | 0 | 0 | 8 (13%) |  |  |
| **Combined Enterobacterales + *P. aeruginosa*** | **931** | **768** | **34** | **129** | **94%** | **98%** | **1 (0.8%)** | **4 (0.5%)** | **47 (5.0%)** |  |  |  |  |  |  |  |  |  |  |  |

Table S4. Data analysis applying 2024 FDA-cleared interpretations to the ASTar BC G- results. Categorical agreement (CA); essential agreement (EA); very major error (VME); major error (ME); minor error (mE); susceptible (S); susceptible dose dependent (SDD); intermediate (I); resistant (R).


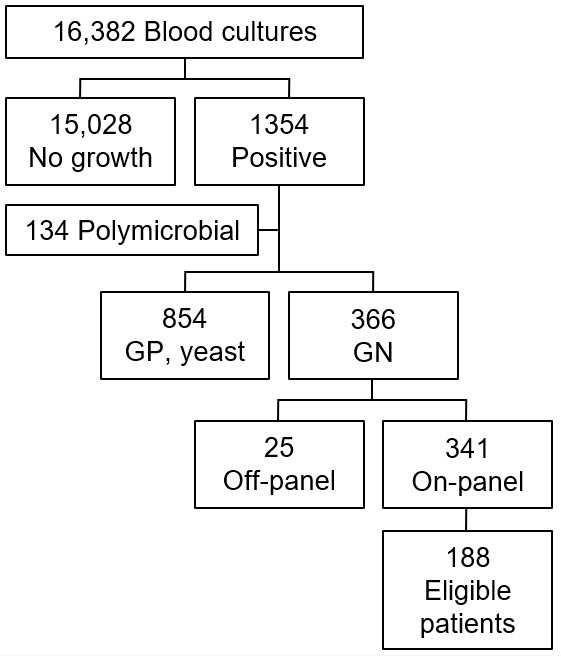


Figure S1. Patients with blood cultures eligible for ASTar from a 6-month period. Eligible patients include those with culture positive for on-panel organisms and the assumption that ASTar would be repeated on unique isolates collected >3 days apart. Gram positive (GP); Gram negative (GN).
